# INFLAMMATORY DISORDERS ASSOCIATED WITH *HELICOBACTER PYLORI* IN THE ROUX-EN-Y BYPASS GASTRIC POUCH

**DOI:** 10.1590/0102-6720201600S10009

**Published:** 2016

**Authors:** Luiz Claudio Lopes CHAVES, Isabela Klautau Leite Chaves BORGES, Maíra Danielle Gomes de SOUZA, Ian Passos SILVA, Lyz Bezerra SILVA, Marcelo Alexandre Prado MAGALHÃES, Allan Herbert Feliz FONSECA, Josemberg Marins CAMPOS

**Affiliations:** Postgradute Program in Biology of Infectious and Parasitic Agents, Institute of Biological Sciences, Federal University of Pará, Belém, PA, Brazil

**Keywords:** Helicobacter pylori, Gastrointestinal diseases, Gastric Bypass, Obesity

## Abstract

**Background:**

The prevalence of Helicobacter pylori in obese candidates for bariatric surgery and its role in the emergence of inflammatory lesions after surgery has not been well established.

**Aim:**

To identify the incidence of inflammatory lesions in the stomach after bariatric surgery and to correlate it with H. pylori infection.

**Methods:**

This is a prospective study with 216 patients undergoing Roux-en-Y gastric bypass. These patients underwent histopathological endoscopy to detect H. pylori prior to surgery. Positive cases were treated with antibiotics and a proton inhibitor pump followed by endoscopic follow-up in the 6^th^ and 12^th^ month after surgery.

**Results:**

Most patients were female (68.1%), with grade III obesity (92.4%). Preoperative endoscopy revealed gastritis in 96.8%, with H. pylori infection in 40.7% (88/216). A biopsy was carried out in 151 patients, revealing H. pylori in 60/151, related to signs of inflammation in 90% (54/60). In the 6^th^ and 12^th^ month after surgery, the endoscopy and the histopathological exam showed a normal gastric pouch in 84% of patients and the incidence of H. pylori was 11% and 16%, respectively. The presence of inflammation was related to H. pylori infection (p<0,001).

**Conclusion:**

H. pylori has a similar prevalence in both obese patients scheduled to undergo bariatric surgery and the general population. There is a low incidence of it in the 6^th^ and 12^th^ months after surgery, probably owing to its eradication when detected prior to surgery. When inflammatory disease is present in the new gastric reservoir it is directly related to H. pylori infection.

## RESUMO

**Racional:**

Helicobacter pylori é responsável por várias doenças gastrointestinais. Com o aumento de cirurgia bariátrica no país, há poucos estudos sobre a prevalência desta bactéria em obesos com indicação cirúrgica e o seu papel no surgimento de lesões inflamatórias no pós-operatório.

**Objetivo -:**

Identificar a incidência de lesões inflamatórias no estômago pós-cirurgia bariátrica e correlacionar com a infecção por H. pylori.

**Métodos -:**

Estudo prospectivo com dois grupos de pacientes. Em ambos os grupos verificou-se a prevalência do H. pylori no pré-operatório através de histopatologia, mas em apenas um dos grupos, nos casos de H. pylori positivo realizou-se o tratamento com antibioticoterapia e inibidor de bomba de próton com realização de nova endoscopia no 6° e 12° mês pós-operatório.

**Resultados:**

Avaliou-se 216 pacientes, com as seguintes características: sexo feminino (68,1%), faixa etária entre 30-40 anos, com 31,9% e 31%, respectivamente. De acordo com o IMC, 17,6% apresentavam obesidade moderada, 82,4% obesidade severa/mórbida e 9,7% superobesidade. Nos pacientes submetidos à endoscopia, a positividade do H. pylori se manifestou em 40,7%, sendo responsável pela atividade inflamatória na mucosa gástrica (p<0,001). No pós-operatório, investigou-se a mucosa gástrica através de endoscopia e histopatologia no 6° e 12° mês, que demonstrou normalidade no neorreservatorio gástrico em 84% dos pacientes, e a incidência de H. pylori foi 11% aos seis meses e 16% aos 12 meses, sendo a presença de processo inflamatório relacionado com a infecção pela bactéria (p<0,001).

**Conclusão:**

- H. pylori apresenta prevalência similar tanto em obesos que irão submeter-se à cirurgia bariátrica quanto à população em geral; há baixa incidência dele no 6° e 12° mês após a operação e isto deve-se provavelmente à sua erradicação quando detectado no pré-operatório; quando presente a doença inflamatória no neorreservatório gástrico possui relação direta com a infecção por H. pylori.

## INTRODUCTION

*.H pylori* infection has an incidence of 24-67% among bariatric patients. Upper gastrointestinal endoscopy (UGE) is used prior to surgery to detect this bacteria, in view of its high incidence and possible relation with pathological abnormalities of the stomach. In some locations, such as Finland, UGE isprerequisite for all bariatric patients, although this practice is still questioned^9^ ^,^ ^14^.

Inflammatory diseases of the stomach after bariatric surgery, especially Roux-en-Y gastric bypass (RYGB), include gastritis and ulcers (of the new gastric reservoir and the anastomosis). There is no difference in the etiopathogeny of these lesions in the operated or non-operated stomach, with *H. pylori* being the main cause and non-steroid anti-inflammatory drugs the secondary cause. However, the relation between these lesions and RYGB is not fully understood^9^ ^,^ ^11^.

UGE to detect *H. pylori* prior to bariatric surgery has been required in triage for the presence of this bacteria by health insurance plans, especially in cases of RYGB. This requirement is based on the supposition that the existence of these bacteria is linked to ulcers or cancers of the excluded stomach after the procedure. The present study uses a number of tests to conclude its diagnosis, including the rapid urease test, histology and tissue biopsy, along with non-endoscopic tests of blood and serum^10^ ^,^ ^13^.

The aim of this study was to identify the incidence of inflammatory lesions in the stomach after bariatric surgery and to correlate it with *H. pylori* infection.

## METHODS

The study was approved by the Research Ethics Committee of the Federal University of Pará (Tropical Medicine Unit), Belém, PA, Brazil. All patients were studied in accordance with the precepts of the Helsinki Declaration and the Nuremberg Code and the norms for research involving human beings were respected (Res. CNS 196/96).

A prospective study was carried out with two groups of patients from the Bariatric Surgery Service of Hospital Porto Dias in Belém, PA, Brazil. The two groups underwent surgical treatment for obesity, in accordance with CFM Resolution No. 1,766/05. 

The first group was used to study the prevalence of *H. pylori* infection in obese patients through histopathological examination of fragments obtained by endoscopic biopsy prior to surgery. Patients testing positive underwent eradication treatment using antibiotics as outlined in the 2^nd^ Brazilian Consensus on the Study of *H. pylori,* using a combination of PPI, clarithromycin and amoxicillin.

The second group was composed of at least 100 patients of the first group, who underwent endoscopy at 6 and 12 months after surgery, in order to evaluate the incidence of bacteria and inflammatory diseases of gastric pouch*.*

## RESULTS

In the first phase of the study, 2010-2012, 216 obese patients indicated for surgery were analyzed. 147/216 (68.1%) were female and 69/216 (31.9%) male; most patients were in the third or fourth decade of life, 69 (31.9%) and 67 (31%) respectively, with progressively fewer in the older age groups and few in the second decade. 

According to BMI, 38/216 (17.6%) were moderately obese, 178/216 (72.7%) severely or morbidly obese and 21/216 (9.7%) super obese. Prior to surgery, the 216 patients underwent upper gastrointestinal endoscopy (UGE), 209 (96.8%) of whom presented with gastritis. The prevalence of *H. pylori* in patients in this group was 88/216 (40.7%), although 128/216 (59.3%) showed no signs of these bacteria.

The prevalence of *H. pylori* by gender was similar for both sexes, 28/88 (40.6%) for men and 60/88 (40.8%) for women and there was not statistically significant correlation (p=0.9736). The distribution by age group and BMI showed a difference although this was not statistically significant (p<0.3114).

Analysis of the presence of inflammatory activity in the gastric mucosa prior to surgery was carried out in 151 of the 216 patients studied. Of these 60/151 tested positive for *H. pylori* and 54/60 (90%) had a histologically active inflammatory process, compared to 26/91 (28.6%) of patients in whom the bacteria was not found, and this was a significant difference (p<0.001). The likelihood of the presence of *H. pylori* among patients with inflammatory activity was 22 times greater than in patients without such activity (OR=22.5, Table 1).

**TABLE 1:** The presence of inflammatory activity in the gastric mucosa and *H. pylori* infection

Histo-Pre	*H. pylori*-Pre			Total
	Negative	%	Positive	%	
No activity	65	71.4	6	10	71
Activity	26	28.6	54	90	80
Total	91	100	60	100	151

Six months after surgery, 109 patients were evaluated and 92 (84.4%) had normal UGE, 15 (13.8%) presented with gastritis and 2 (1.8%) ulcer of the new reservoir. Including patients with gastritis and ulcer 15.6% presented with inflammatory disease.

The test for *H. pylori* found 13 (11.9%) of these 109 patients to be positive and 96 (88.1%) negative.

Among the 92 patients with normal endoscopy six months after surgery, the incidence of *H. pylori* was 7 (7.6%), while *H. pylori* was present in 6 (35.3%) of the 17 patients with endoscopic gastritis (p<0.0047). The likelihood of *H. pylori* being present in the patients with gastritis was six times greater than among patients without gastritis (OR=6, Table 2).

**TABLE 2:** Correlation between results of endoscopy and *H.pylori* infection six months after surgery

Endoscopy(6 months)	H. pylori - 6 months				Total
	Negative	%	Positive	%	
Normal	85	88.5	7	53.8	92
Gastritis	11	11.5	6	46.2	17
Total	96	100	13	100	109

**FIGURE 1:**
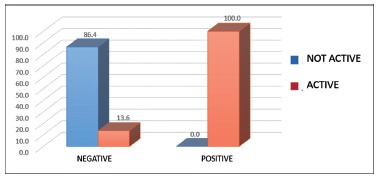
Analysis of patients regarding the presence of inflammatory activity in the mucosa of the new gastric reservoir and *H. pylori* infection.

Histological analysis after six months was carried out in 54 patients to investigate the presence of inflammatory activity in the gastric mucosa in this group and *H. pylori* was found to be present in all nine patients with this activity (100%, p<0.0001, Figure 1).

In the group of patients evaluated 12 months after surgery, 125 underwent UGE, of whom 105 (84%) presented with a normal endoscopy, 15 (12%) with gastritis and 5 (4%) with an ulcer in the new reservoir. Including gastritis and ulcers, there were 20 (16%) cases of inflammatory disease in the operated stomach. 

Of these 125 patients, 19 (15.2%) tested positive for *H. pylori* and 106 (84.8%) negative (Table 3).

**TABLE 3:** Results for *H. pylori* infection in patients 12 months after surgery

H. pylori	Cases	%
Negative	106	84.8
Positive	19	15.2
Total	125	100

Of the 105 patients with normal endoscopy 12 months after surgery, *H. pylori* was found in 14 (13.33%), while, in the 20 patients with endoscopic gastritis, *H. pylori* was present in 5 (25%, p<0.3211,Table 4).

**TABLE 4:** Correlation between results of endoscopy and *H.pylori* infection 12 months after surgery

Endoscopy (12 months)	H. pylori - 12 months				Total
	Negative	%	Positive	%	
Normal	91	85.8	14	73.7	105
Gastritis	15	14.2	5	26.3	20
TOTAL	106	100	19	100	125

Analysis of the correlation between *H. pylori* and inflammation of the gastric mucosa showed that 17 of the 59 patients undergoing endoscopic biopsy (28.81%) had inflammatory activity and nine of these tested positive for *H. pylori*, compared to two among the 40 normal examinations (p<0.0001). the likelihood of *H. pylori* being present in patients with histological inflammatory activity was 22 times greater than in patients without this activity (OR=22.5) (Table 5).

**TABLE 5:** Analysis of patients regarding the presence of inflammatory activity in the mucosa of new gastric reservoir and *H. pylori* infection

Histo (12 months)	H. pylori - 12 months				Total
	Negative	%	Positive	%	
No activity	40	83.3	2	18.2	42
Activity	8	16.7	9	81.8	17
TOTAL	48	100	11	100	59

There were no statistically significant alterations in the incidence of *H. pylori* for the variables age, gender, or BMI. 

## DISCUSSION

There are divergences in the literature as to the prevalence of *H. pylori* in the obese. In Saudi Arabia, it is found in 68-82.2% of the population and is attributed to socioeconomic and sanitary factors. In the obese, the bacteria was present in 85.5% of patients who have undergone bariatric surgery^1^.

A systematic review has shown that the prevalence of *H. pylori* in obese patients scheduled to undergo bariatric surgery varies from 6.9-61.3%. The prevalence of infection caused by this pathogen varies from 30 to 90% around an average of 60%^7^.

In a national study, the prevalence of *H. pylori* was 60%. The authors recommended the use of two methods to research the bacteria (urease and histology) to increase accuracy^2^.

RYBG surgery involves resection a part of the stomach that is called the excluded stomach. This stomach has a high probability of developing abnormalities that may be the consequence of bile and pancreatic secretion reflux. *H. pylori* may be one of the causes of some dysfunctions and should be treated with caution prior to surgery, since the exclusion of this part of the stomach makes access to it difficult^16^.

*H. pylori* infection causes inflammation of the gastric mucosa and may lead to problems such as intestinal metaplasia and even cancer. Its eradication may revert this inflammatory process but this is not possible in more advanced phases^7^. 

The need for endoscopy prior to surgery is still controversial. In a study conducted by Wong *et al*. with 180 patients undergoing gastric bypass, an alarming number of 159 were diagnosed with chronic superficial gastritis and esophageal reflux, erosion, hiatal hernia and gastric ulcer were also found in smaller numbers of patients^18^.

In a recent literature review Palermo *et al*. showed that the presence of *H. pylori* prior to surgery may be related to the development of postoperative marginal ulceration. Thus, patients with upper gastrointestinal symptoms should undergo endoscopy prior to gastric bypass and be treated for *H. pylori* if they test positive. However, some authors believe that the prevalence in patients undergoing RYGB is similar to that of the general patient and that the *H. pylori* test and preoperative treatment do not diminish the incidence of anastomotic ulcer or gastritis in the gastric pouch^12^.

Apart from UGE, a biopsy is also fundamental in determining the future management of the surgical procedure and may shift it to initial treatment of an existing pathological abnormality. *H. pylori* is already known to be a carcinogenic agent, which operates by way of chronic gastritis or intestinal metaplasia. These changes in the stomach undergoing RYGB surgery may be harmful, because of the existence of the excluded stomach, leading to serious complications, if abnormalities are not identified prior to the procedure^6^ ^,^ ^7^.

Considering the possible endoscopic alterations found in the UGE on patients undergoing bariatric surgery, research suggests a classification of endoscopic findings in the preoperative RYGB, reinforcing the importance of preoperative screening^5^.

In the present study of 216 patients, the prevalence of *H. pylori* was 40.7% and there were no statistically significant differences in terms of sex, age group or BMI. Histopathological analysis of the mucosa proved to be significant (p<0.001) with the bacteria responsible for inflammatory activity in 90%. In a study of 854 patients undergoing bariatric surgery, the prevalence of *H. pylori* was around 23.7%, but the article cites other sources giving a range of prevalence of the bacteria that varies from 11.5-66.7%^17^.

The present study eradicated *H. pylori* in patients who had tested positive for the bacteria prior to surgery. Treatment followed the schema outlined in the 3^rd^ Brazilian *Helicobacter pylori* Consensus, with an eradication rate of nearly 80%^4^ ^,^ ^8^. The 109 patients who had undergone RYGB six months earlier were symptomless and 84.4% of these presented with a normal endoscopy and 15.6% with inflammatory disease of the new reservoir. The incidence of *H. pylori* in patients was 11.9%, but the incidence in those with inflammatory disease of the new reservoir was 35.3% (p<0.004). 

The likelihood of testing positive for *H. pylori* in patients with inflammatory disease of the new reservoir is six times greater than in those without inflammatory disease, strongly indicating a relation between the presence of the bacteria and inflammatory lesions of the operated stomach. However, there is controversy in the literature regarding the presence of this pathogen and inflammatory lesions. Rawlins *et al*. showed, in 228 patients undergoing RYGB, that there was no evidence of a connection between *H. pylori* and an increase in the postoperative complications rate, further underlining the importance of this study in scientific circles^15^.

This becomes even more apparent when the histopathological exams of these patients are taken into consideration. Cross-tabulation of the presence of inflammatory activity of the gastric mucosa with the presence of *H. pylori*, showed that nine of the 53 exams conducted revealed active inflammatory activity and all showed infection with *H. pylori* (p< 0.001).

In the 12^th^ month after surgery, 125 symptomless patients were evaluated and 84% had normal endoscopy, while 16% had inflammatory disease of the new reservoir. *H. pylori* was present in 15.2%, a little higher than the incidence in the 6^th^ month after surgery but without statistical significance (p<0.3211).

Analysis of the gastric mucosa of 59 patients after 12 months revealed 17 with inflammatory activity, nine of whom tested positive for *H. pylori,* compared to two of the 42 histopathological exams with absence of inflammatory activity (p<0.001). The likelihood of *H. pylori* being present in patients with inflammatory activity was 22 times greater than in patients without such activity, clearly indicating the relation between inflammatory disease of the new gastric reservoir and *H. pylori* infection.

In the patients studied, the low incidence of ulceration of the gastric stump and gastritis may be related to routine eradication of *H. pylori* in our protocol. Furthermore, other studies have shown that eradication of *H. pylori* may be related to a decrease in the incidence of perforations of the viscera and postoperative marginal ulcers. In one study of 560 patients, the incidence of ulceration was 2.4% in tested and treated patients, compared to 6.8% in another study where this protocol was not applied^3^.

 The study did not include a test after treatment to confirm eradication of *H. pylori* in the 6^th^ and 12^th^ month after surgery, given the failure rate of around 10% for the classical treatment. It is thus important to note that patients testing positive for *H. pylori* after surgery need to have their data cross-tabulated with preoperative data to evaluate whether they were already positive and, in this case, to opt for second line treatment, thereby avoiding failure for reason of bacterial resistance. 

Research into both *H. pylori* and possible lesions of the excluded stomach poses a challenge for scientific studies, owing to the possible emergence of overwhelming technical difficulties, sometimes making it impossible to conduct the procedure. The difficulty is not restricted to research but also impedes treatment^7^. 

The results obtained by the present study indicate the importance of diagnosis of the presence of *H. pylori* in patients undergoing bariatric surgery, especially when the RYGB technique is used*,* since this technique involves excluding part of the stomach, which may lead to the emergence of inflammatory diseases in the new gastric reservoir.

## CONCLUSIONS

*H. pylori* has a similar prevalence in both obese patients scheduled to undergo bariatric surgery and the general population. There is a low incidence of it in the 6^th^ and 12^th^ months after surgery, probably owing to its eradication when detected prior to surgery. When inflammatory disease is present in the new gastric reservoir it is directly related to *H. pylori* infection.
